# Use of Guideline-Based Therapy for Diabetes, Coronary Artery Disease, and Chronic Kidney Disease After Acute Kidney Injury: A Retrospective Observational Study

**DOI:** 10.1177/20543581221103682

**Published:** 2022-06-14

**Authors:** Sunchit Madan, Patrick A. Norman, Ron Wald, Javier A. Neyra, Alejandro Meraz-Muñoz, Ziv Harel, Samuel A. Silver

**Affiliations:** 1 Division of Nephrology, St. Joseph’s Healthcare Hamilton, McMaster University, Hamilton, ON, Canada; 2 Kingston General Health Research Institute, Kingston, ON, Canada; 3 Department of Public Health Sciences, Queen’s University, Kingston, ON, Canada; 4 Division of Nephrology, St. Michael’s Hospital, University of Toronto, ON, Canada; 5 Division of Nephrology, Bone and Mineral Metabolism, Department of Internal Medicine, University of Kentucky, Lexington, USA; 6 Division of Nephrology, Kingston Health Sciences Centre, Queen’s University, Kingston, ON, Canada

**Keywords:** acute kidney injury, cardiovascular diseases, cardiovascular risk, ACE inhibitors, angiotensin receptor blocker

## Abstract

**Background:**

Survivors of acute kidney injury (AKI) are at a high risk for cardiovascular complications. An underrecognition of this risk may contribute to the low utilization of relevant guideline-based therapies in this population.

**Objective:**

We sought to assess accordance with guideline-based recommendations for survivors of AKI with diabetes, coronary artery disease (CAD), and preexisting chronic kidney disease (CKD) in a post-AKI clinic, and identify factors that may be associated with guideline accordance.

**Design:**

Retrospective cohort study.

**Setting:**

Post-AKI clinics at 2 tertiary care centers in Ontario, Canada.

**Patients:**

We included adult patients seen in both post-AKI clinics between 2013 and 2019 who had at least 2 clinic visits within 24 months of an index AKI hospitalization.

**Measurements:**

We assessed accordance to recommendations from the most recent North American and international guidelines available at the time of study completion for diabetes, CAD, and CKD.

**Methods:**

We compared guideline accordance between visits using the Cochran Mantel Haenszel test. We used multivariable Poisson regression to identify prespecified factors associated with accordance.

**Results:**

Of 213 eligible patients, 192 (90%) had Kidney Disease Improving Global Outcomes Stage 2-3 AKI, 91 (43%) had diabetes, 76 (36%) had CAD, and 88 (41%) had preexisting CKD. From the first clinic visit to the second, there was an increase in angiotensin-converting enzyme inhibitor/angiotensin receptor blocker (ACE-I/ARB) use across all disease groups—from 33% to 46% (*P* = .028) in patients with diabetes, from 30% to 57% (*P* = .002) in patients with CAD, and from 16% to 35% (*P* < .001) in patients with preexisting CKD. Statin use increased in patients with preexisting CKD from 64% to 71% (*P* = .034). Every 25 μmol/L rise in the discharge serum creatinine was associated with a 19% (95% confidence interval [CI], 8%-28%) and 12% (95% CI, 2%-21%) lower likelihood of being on an ACE-I/ARB in patients with diabetes and preexisting CKD, respectively.

**Limitations:**

The study lacked a comparison group that received usual care. The small sample and multiple comparisons make false positives possible.

**Conclusion:**

There is room to improve guideline-based cardiovascular risk factor management in survivors of AKI, particularly ACE-I/ARB use in patients with an elevated discharge serum creatinine.

## Abrégé

**Contexte:**

Les survivants d’un épisode d’insuffisance rénale aiguë (IRA) courent un risque élevé de subir des complications cardiovasculaires. Une sous-reconnaissance de ce risque pourrait contribuer à la faible utilisation des thérapies pertinentes recommandées par les lignes directrices dans cette population.

**Objectif:**

Évaluer la conformité aux recommandations des lignes directrices pour les survivants d’un épisode d’IRA souffrant de diabète, de maladie coronarienne et d’insuffisance rénale chronique (IRC) préexistante dans une clinique post-IRA, et identifier les facteurs pouvant être associés à la conformité aux recommandations.

**Conception:**

Étude de cohorte rétrospective.

**Cadre:**

Les cliniques post-IRA de deux centres de soins tertiaires de l’Ontario (Canada).

**Patients:**

Nous avons inclus les patients adultes suivis dans les deux cliniques post-IRA entre 2013 et 2019 et ayant visité la clinique au moins deux fois dans les 24 mois suivant une hospitalisation pour IRA.

**Mesures:**

Nous avons évalué la conformité aux recommandations des plus récentes lignes directrices nord-américaines et internationales disponibles pour le diabète, la maladie coronarienne et l’IRC au terme de l’étude.

**Méthodologie:**

Nous avons comparé la conformité aux recommandations entre les visites à l’aide du test Cochran Mantel Haenszel. La régression multivariée de Poisson a servi à établir les facteurs préspécifiés associés à la conformité.

**Résultats:**

Sur les 213 patients admissibles, 192 (90 %) présentaient une IRA de stade KDIGO 2-3, 91 (43 %) étaient diabétiques, 76 (36 %) présentaient une maladie coronarienne et 88 (41 %) une IRC préexistante. Entre la première et la deuxième visite à la clinique, l’utilisation des inhibiteurs de l’enzyme de conversion de l’angiotensine/antagonistes des récepteurs de l’angiotensine (IECA/ARA) a augmenté dans tous les groupes, soit de 33 à 46 % (*p* = 0,028) chez les diabétiques, de 30 à 57 % (*p* = 0,002) chez les patients souffrant de maladie coronarienne et de 16 à 35 % (*p* < 0,001) chez ceux qui avaient une IRC préexistante. L’utilisation des statines est passée de 64 à 71 % (*p* = 0,034) chez les patients avec une IRC préexistante. Chaque augmentation de 25 μmol/L de la créatinine sérique à la sortie de l’hôpital a été associée, chez les diabétiques et les patients avec une IRC préexistante, à une diminution de la probabilité d’être sous IEAC/ARA de 19 % (IC 95 %: 8-28 %) de 12 % (IC 95 %: 2-21 %) respectivement.

**Limites:**

L’étude ne comportait pas de groupe témoin recevant les soins habituels. Le faible échantillon et les multiples comparaisons rendent possibles les faux positifs.

**Conclusion:**

Il est possible d’améliorer la prise en charge fondée sur les lignes directrices des facteurs de risques cardiovasculaires chez les survivants d’un épisode d’IRA; particulièrement l’utilisation des IECA/ARA chez les patients dont la mesure de créatinine sérique est élevée à la sortie de l’hôpital.

## Introduction

Acute kidney injury (AKI) affects almost 1 in 4 hospitalized patients,^1^ and its incidence is increasing.^2^ Patients who survive an episode of AKI remain at risk for poor short- and long-term outcomes, including cardiovascular disease (CVD), new or worsening chronic kidney disease (CKD), and death.^3-5^ However, these risks are underappreciated by patients and health care providers. For example, less than half of hospital discharge summaries communicate the occurrence of AKI,^6,7^ and most patients are unaware of their AKI diagnosis.^8^ Furthermore, only 10% to 20% of survivors of AKI see a nephrologist within 1 year of hospital discharge.^9-11^

This underrecognition and corresponding lack of follow-up care may contribute to the underutilization of guideline-based therapy for survivors of AKI with diabetes, coronary artery disease (CAD), and preexisting CKD.^12-14^ This care gap is important because recent observational data suggest improved outcomes with the use of angiotensin-converting enzyme inhibitors (ACE-I) or angiotensin receptor blockers (ARB), and statins after AKI of all severities.^15,16^ It is also possible that the mortality reduction observed with nephrologist follow-up in patients with AKI requiring kidney replacement therapy is attributable to more awareness and attention devoted to evidence-based cardiovascular risk reduction strategies.^17,18^

In our nephrologist-led post-AKI clinics, we sought to examine how cardiovascular risk factors are managed after AKI. Our objectives were the following: to assess accordance with guideline-based recommendations for survivors of AKI with diabetes, CAD, and preexisting CKD in a post-AKI clinic; to identify factors which may affect guideline accordance in this population; and to inform future quality improvement initiatives directed at medication use after AKI.

## Methods

### Post-AKI Clinic Population and Study Design

This is a retrospective cohort study using the post-AKI clinic databases from Kingston Health Sciences Centre and St. Michael’s Hospital. Both hospitals are tertiary care centers located in Ontario, Canada, that have similar post-AKI clinic models.^19^ These post-AKI clinics follow patients who are discharged from hospital after Kidney Disease Improving Global Outcomes (KDIGO) stages 2-3 AKI and/or have non-recovery of their baseline kidney function (defined as having a discharge serum creatinine ≥ 25% of the pre-AKI baseline). Patients may be referred by any hospital health care provider. These clinics do not follow patients who already receive outpatient nephrology follow-up as part of standard practice, such as those with a baseline estimated glomerular filtration rate (eGFR) ≤15 mL/min/1.73 m^2^; persistent requirement for kidney replacement therapy (KRT); a functioning kidney transplant; or a clinical suspicion of glomerulonephritis, polycystic kidney disease, myeloma cast nephropathy, or thrombotic microangiopathy.

Each visit to the post-AKI clinic consists of a standardized assessment that emphasizes blood pressure (BP) and proteinuria control, volume assessment, cardiovascular risk reduction, management of CKD complications, and detailed medication reviews with emphasis on diuretics and cardioprotective drugs (Supplementary Document 1). Nephrologists are also free to tailor treatment to individual patient needs. The AKI clinics aim to schedule all initial consultation appointments within 90 days of hospital discharge (as per KDIGO recommendations),^20^ with the frequency of subsequent follow-up at the nephrologist’s discretion. Due to competing health demands and patient travel requirements, some delays do occur resulting in the first clinic visit sometimes being beyond the 90-day target.

For this study, we identified all patients aged 18 years and older who attended the post-AKI clinic between 2013 and 2019. To assess guideline accordance in the post-AKI clinic over time, we required patients to have a minimum of 2 clinic visits. The first clinic visit had to occur within 365 days of discharge from the index AKI hospitalization, and the second visit had to occur 20 to 365 days after the first visit. If patients had multiple follow-up visits during this timeframe, we preferentially selected the follow-up visit that was closest to 270 days after the first clinic visit (as this visit would be approximately 12 months after hospital discharge). Hereafter, this visit is referred to as the “second eligible visit.” This decision ensured the final assessment of guideline-based targets was made between 12 and 24 months after the index hospitalization, giving ample opportunity for patients to receive risk reduction strategies.

The Research Ethics Boards at both participating sites approved the study, which adhered to the Declaration of Helsinki. Reporting of the study follows the Strengthening the Reporting of Observational Studies in Epidemiology (STROBE) guidelines.^21^

### Outcomes and Definition of Guideline-Based Therapy

The outcomes of interest were in accordance with guideline-based recommendations for the management of patients with diabetes, CAD (defined by a previous acute coronary event and/or percutaneous coronary intervention or coronary artery bypass graft), and preexisting CKD (defined as baseline eGFR <60 mL/min/1.73 m^2^, with baseline serum creatinine determined by the most recent outpatient value between 7 and 365 days prior to hospital admission).^22^ Proteinuria was not part of the definition of preexisting CKD. We used the recommendations from the most recent North American and international guidelines available at the time of study completion that had Level A or B evidence, with some modifications based on data availability. These definitions included the following:

1. For patients with diabetes^23^:
  - BP ≤ 130/80 mm Hg; statin therapy; metformin therapy (all eGFR values eligible); ACE-I/ARB therapy in those with CVD (defined by the presence of one or more of hypertension, peripheral vascular disease, CAD and/or previous episode of a cerebrovascular accident).
2. For patients with CAD^24^:
  - BP ≤ 140/90 mm Hg; statin therapy; aspirin therapy; beta-blocker therapy; ACE-I/ARB therapy.
3. For patients with preexisting CKD^25,26^:
  - BP ≤ 140/90 mm Hg; statin therapy; ACE-I/ARB therapy.

We determined medication usage via self-reporting by patients at clinic visits. All patients either brought their medications with them to the clinic, or had their prescriptions verified with a pharmacist. We measured BP using a BpTRU machine, which takes serial electronic readings and provides an average of these as the final value, complying with Hypertension Canada’s recommendations on office BP measurements.^27^ As the most recent hypertension guidelines were published after the study’s completion, the recommendation for targeting a systolic BP of less than 120 mm Hg was not used for our study.

We assessed utilization of guideline-based therapies at both clinic visits. Patients with multiple comorbidities could qualify for overlapping recommendations. For example, we assessed patients with diabetes and preexisting CKD for treatment with an ACE-I/ARB, statin, and metformin, as well as having a BP target ≤130/80 mm Hg (ie, the lowest applicable BP target). We also calculated a composite score to act as a summary measure of overall treatment. We defined this as the total number of interventions performed among eligible patients, divided by the total number of possible non-overlapping interventions among eligible patients. For example, patients with diabetes and preexisting CKD qualify for 4 recommendations (ACE-I/ARB, metformin, statin, and BP≤130/80 mm Hg). If such patient is only on metformin and a statin, the numerator would be 2 (number of met recommendations) and the denominator would be 4 (number of non-overlapping eligible recommendations). Therefore, the composite score quantifies overall performance into a single variable, which has been used in other similar studies.^28,29^

### Statistical Analysis

We summarized baseline characteristics for the overall cohort and stratified by disease groups (diabetes, CAD, and preexisting CKD). We presented categorical variables as counts with percentages and continuous variables as means and standard deviations or medians and interquartile ranges.

To evaluate whether the timing of nephrologist follow-up (ie, first or second eligible clinic visit) was related to guideline accordance, we compared usage of guideline-based therapy between the 2 clinic visits using the Cochran Mantel Haenszel test, accounting for within-patient correlation between repeated observations. For the composite score, we compared usage of guideline-based therapy between the 2 clinic visits with a paired *t* test. We used Poisson regression with robust standard errors to identify whether age, sex, baseline serum creatinine, discharge serum creatinine, and eligible clinic visit number (first clinic visit = within 365 days of discharge; second eligible visit = 20-365 days after the first clinic visit) were independently associated with the use of guideline-based therapy at any time over the follow-up period. For the composite score, we used generalized estimating equations with a linear link function, normal distribution, and unstructured covariance to account for repeated observations within patients. We also added diabetes, CAD, and KDIGO AKI stage to these models.

All statistical analyses were performed using SAS software version 9.4 and SAS/STAT software version 14.2 (SAS Institute, Cary, North Carolina). All *P* values were 2-tailed, with the threshold for statistical significance set at .05.

## Results

### Patient Characteristics

**Figure 1.**
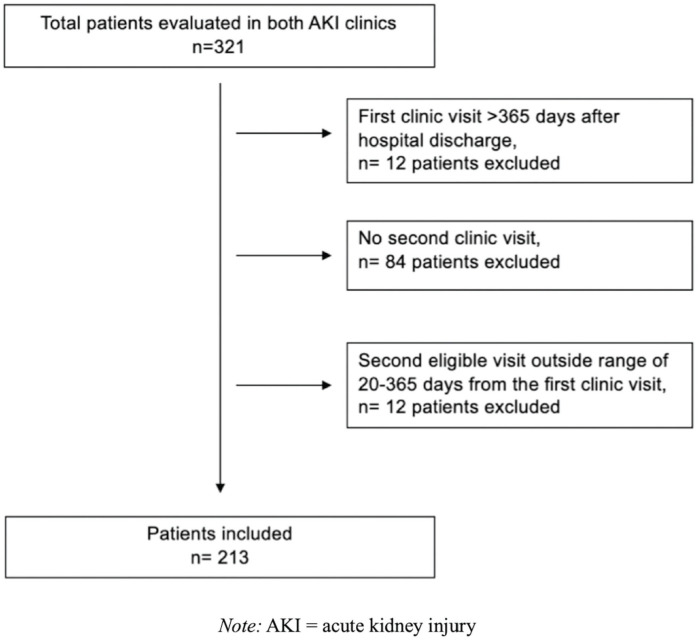
Cohort of survivors of AKI identified for the study using the defined inclusion and exclusion criteria.

We identified 321 patients seen in both post-AKI clinics. We excluded 12 patients who had their first clinic visit more than 365 days from hospital discharge, 84 patients who did not have a second eligible visit, and 12 patients who did not have a second eligible visit that was 20 to 365 days after the first clinic visit (Figure 1). The median time from hospital discharge to the first clinic visit was 40.0 [22.0-70.0] days, and the median time from the first visit to the second eligible visit was 228.0 [168.0-274.0] days.

Of the 213 eligible patients (Table 1), 91 (43%) patients had diabetes, 76 (36%) had CAD, and 88 (41%) had preexisting CKD. The mean age was 65.7 ± 14.1 years, 141 (66%) were male, and the mean baseline eGFR was 68.8 ± 24.6 mL/min/1.73 m^2^. In the CKD subgroup, the mean baseline eGFR was 45.4 ± 9.2 mL/min/1.73 m^2^.

**Table 1.** Baseline Characteristics of Study Participants, Stratified by Comorbidity.

Baseline characteristics	Full cohort (n = 213)	Diabetes (n = 91)	CAD (n = 76)	CKD (n = 88)
Age (years) mean ± SD	65.7 ± 14.1	67.5 ± 9.5	71.2 ± 10.3	71.6 ± 10.6
Male, n (%)	141 (66.2)	56 (61.5)	57 (75.0)	43 (48.9)
Ethnicity, n (%)				
White	138 (64.8)	58 (63.7)	49 (64.5)	70 (79.5)
South Asian	18 (8.5%)	10 (11.0%)	7 (9.2%)	3 (3.4%)
Black	9 (4.2%)	4 (4.4%)	4 (5.3%)	3 (3.4%)
Other	43 (20.2%)	19 (20.9%)	15 (19.7%)	11 (12.5%)
Unknown	5 (2.3%)	0	1 (1.3%)	1 (1.1%)
Baseline serum creatinine (μmol/L), mean ± SD^a^	98.3 ± 29.1	98.4 ± 29.4	97.1 ± 27.8	123.6 ± 24.0
Baseline eGFR (mL/min/1.73 m^2^), mean ± SD	68.8 ± 24.6	66.5 ± 22.9	67.8 ± 21.5	45.4 ± 9.2
Baseline proteinuria, n (%)^b^				
Missing	140 (65.7)	39 (42.9)	45 (59.2)	53 (60.2)
Mild/none	28 (13.1)	17 (18.7)	10 (13.2)	15 (17.0)
Moderate	32 (15.0)	24 (26.4)	14 (18.4)	12 (13.6)
Heavy	13 (6.1)	11 (12.1)	7 (9.2)	8 (9.1)
Heart failure, n (%)	58 (27.2)	29 (31.9)	22 (28.9)	29 (33.0)
CAD, n (%)	76 (35.7)	40 (44.0)	76 (100.0)	31 (35.2)
CVA, n (%)	25 (11.7)	11 (12.1)	9 (11.8)	14 (15.9)
PVD, n (%)	40 (18.8)	21 (23.1)	21 (27.6)	17 (19.3)
Diabetes, n (%)	91 (42.7)	91 (100.0)	40 (52.6)	42 (47.7)
Hypertension, n (%)	144 (67.6)	74 (81.3)	61 (80.3)	68 (77.3)
Hyperlipidemia, n (%)	111 (52.1)	60 (65.9)	59 (77.6)	52 (59.1)
Cancer, n (%)	28 (13.1)	11 (12.1)	9 (11.8)	14 (15.9)
Dementia, n (%)	9 (4.2)	3 (3.3)	4 (5.3)	7 (8.0)
Smoking history, n (%)	102 (47.9)	43 (47.3)	43 (56.6)	43 (48.9)
Length of hospital stay (days), median (IQR)	12.0 [6.0-22.0]	10.5 [6.0-22.0]	10.0 [5.5-19.5]	9.0 [4.0-16.0]
ICU stay required, n (%)	107 (50.2)	41 (45.1)	41 (53.9)	35 (39.8)
KDIGO AKI stage, n (%)				
1	21 (9.9)	8 (8.8)	9 (11.8)	19 (21.6)
2	71 (33.3)	36 (39.6)	37 (48.7)	30 (34.1)
3	121 (56.8)	47 (51.6)	30 (39.5)	39 (44.3)
Inpatient KRT required, n (%)	47 (22.1)	16 (17.6)	11 (14.5)	17 (19.3)
Time from discharge to first clinic visit (days), median (IQR)	40.0 [22.0-70.0]	42.0 [20.0-72.0]	41.0 [22.0-66.0]	41.0 [21.0-74.0]
Time from first clinic visit to second eligible visit (days), median (IQR)	228.0 [168.0-274.0]	226.0 [154.0-273.0]	227.0 [173.5-278.0]	238.5 [172.5-289.0]
Discharge serum creatinine (μmol/L), mean ± SD	166.2 ± 90.7	165.9 ± 80.5	146.8 ± 54.0	186.4 ± 76.5
Serum creatinine at first clinic visit (μmol/L), mean ± SD	136.1 ± 60.0	142.3 ± 61.9)	136.1 ± 52.1	161.3 ± 60.3
Serum creatinine at second eligible visit (μmol/L), mean ± SD	129.9 ± 48.3	141.1 ± 54.3	131.5 ± 44.7	149.9 ± 46.8
First Clinic visit eGFR (mL/min/1.73 m^2^), mean ± SD	52.2 ± 25.6	47.8 ± 22.3	49.3 ± 20.9	36.1 ± 12.2
Second eligible visit eGFR(mL/min/1.73 m^2^), mean ± SD	52.9 ± 23.6	47.0 ± 20.7	49.7 ± 20.4	38.5 ± 12.4

*Note.* SD = standard deviation; AKI = acute kidney injury; eGFR = estimated glomerular filtration rate; CAD = coronary artery disease; CVA = cerebrovascular accident; PVD = peripheral vascular disease; IQR = interquartile range; ICU = intensive care unit; KRT = kidney replacement therapy; KDIGO = Kidney Disease Improving Global Outcomes; CKD = chronic kidney disease.

a Defined as the most recent outpatient serum creatinine between 7 and 365 days prior to hospital admission.

b Defined as the most recent outpatient value between 7 and 365 days prior to hospital admission, using a hierarchical combination of albumin or protein to creatinine ratio (ACR or PCR) or urinalysis that has been described previously.^30^ Severe proteinuria was defined as ACR >30 mg/mmol, PCR >50 mg/mmol, or urinalysis protein of 2+ or more (≥100 mg/dL). Moderate proteinuria was defined as ACR between 3 and 30 mg/mmol, PCR between 15 and 50 mg/mmol, or urinalysis protein of trace or 1+ (10-30 mg/dL). Normal/mild proteinuria was defined as ACR < 3 mg/mmol, PCR < 15 mg/mmol, or a normal urinalysis (<10 mg/dL).

During the index AKI hospitalization, 192 (90%) patients had KDIGO stage 2-3 AKI, 47 (22%) patients received KRT, and 143 (67%) patients received an inpatient nephrology consult. The median length of hospital stay was 12.0 [6.0-22.0] days and the mean discharge serum creatinine was 166.2 ± 90.7 μmol/L. The mean systolic and diastolic BP at the first clinic visit was 125.8 ± 21.4 mm Hg and 72.6 ± 13.1 mm Hg, respectively, and the mean serum creatinine was 136.1 ± 60.0 μmol/L. At the second eligible visit, the mean systolic and diastolic BP was 127.0 ± 19.8 mm Hg and 72.6 ± 12.4 mm Hg, respectively, with a mean serum creatinine of 129.9 ± 48.3 μmol/L.

### Use of Guideline-Based Therapy

The mean number of eligible recommendations per patient was 4.0 ± 0.8 in 162 unique patients (ie, when considering patients with multiple comorbidities). The remaining 51 participants (of the total 213) did not have eligible recommendations as they did not have any relevant comorbidities. Based on the composite score, the proportion of eligible recommendations completed increased from 49% ± 27% to 53% ± 29% between visits (*P* = .04).

At the first clinic visit (Table 2), all eligible recommendations for a comorbidity group were satisfied (ie, perfect guideline accordance) in 8 (9%) patients with diabetes, 12 (16%) patients with CAD, and 6 (7%) patients with preexisting CKD. Statin use was above 60% in all disease groups and BP target achievement ranged from 47% to 68%. ACE-I/ARB therapy was used in 28 (33%) patients with diabetes, 23 (30%) patients with CAD, and 14 (16%) patients with preexisting CKD.

**Table 2.** Use of Guideline-Based Therapy in an AKI Follow-up Clinic, Stratified by Comorbidity.

	Diabetes (n = 91)			Coronary artery disease (n = 76)			Chronic kidney disease (n = 88)		
Guideline-based recommendations	First clinic visit	Second eligible visit	*P* value	First clinic visit	Second eligible visit	*P* value	First clinic visit	Second eligible visit	*P* value
Metformin, n (%)	33 (36%)	36 (40%)	0.47						
Aspirin, n (%)				58 (76%)	55 (72%)	0.41			
Statin, n (%)	65 (71%)	71 (78%)	0.058	67 (88%)	68 (90%)	0.71	56 (64%)	62 (71%)	0.034
ACE-I/ARB, n (%)	28 (33%)	39 (46%)	0.028	23 (30%)	43 (57%)	0.002	14 (16%)	31 (35%)	<.0001
Beta-Blocker, n (%)				64 (84%)	56 (74%)	0.011			
BP target, n (%)	43 (47%)	46 (51%)	0.60	52 (68%)	53 (70%)	0.858	58 (66%)	59 (67%)	0.866
Perfect accordance, n (%)^a^	8 (9%)	10 (11%)	0.56	12 (16%)	17 (22%)	0.2	6 (7%)	18 (21%)	0.001

*Note.* The median time from hospital discharge to the first clinic visit was 40.0 [22.0-70.0] days, and the median time from the first visit to the second eligible visit was 228.0 [168.0-274.0] days. ACE-I/ARB = angiotensin-converting enzyme inhibitor/angiotensin receptor blocker; BP = blood pressure.

a Perfect accordance: defined as meeting all eligible recommendations for a comorbidity group.

From the first clinic visit to the second eligible visit, there was a significant increase in the use of ACE-I/ARB therapy across all disease groups. It increased from 33% to 46% (*P* = .028) in patients with diabetes, from 30% to 57% (*P* = .002) in patients with CAD, and from 16% to 35% (*P* < .001) in patients with preexisting CKD. For most of the other recommendations, use of guideline-based therapy increased between visits but did not reach statistical significance. The exceptions were patients with CAD where the usage of beta-blockers decreased from 84% to 74% (*P* = .01), as well as the preexisting CKD subgroup where statin usage increased from 64% to 71% (*P* = .034) and perfect guideline accordance increased from 7% to 21% (*P* = .001).

### Factors Associated With the Use of Guideline-Based Therapy

**Figure 2.**
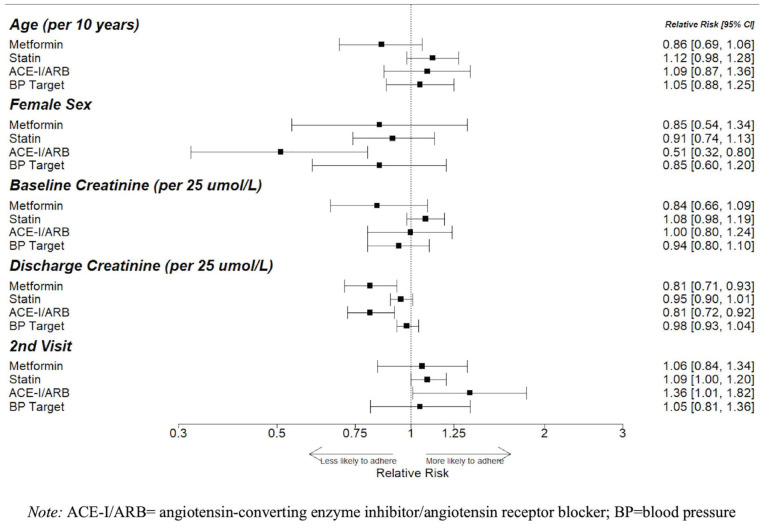
Factors associated with guideline accordance in patients with diabetes. Relative risks (RRs) and 95% confidence intervals (95% CI) reflecting the size of these associations are shown; 95% CIs that do not cross 1 denote statistical significance.

**Figure 3.**
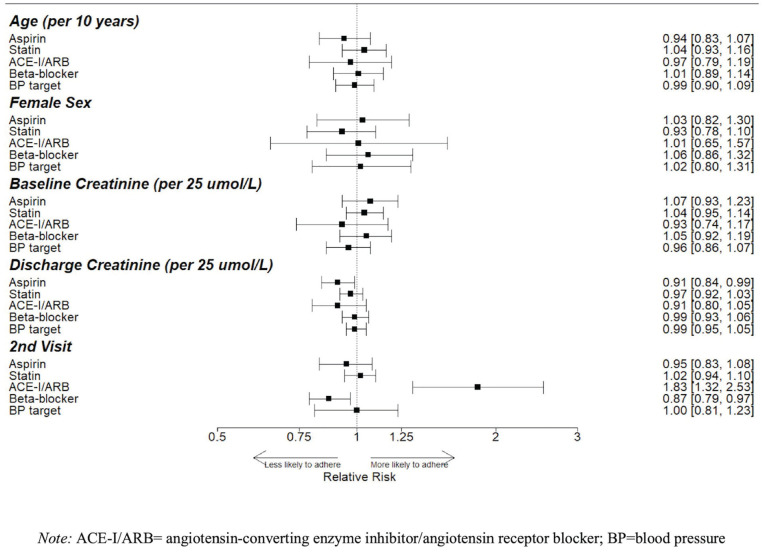
Factors associated with guideline accordance in patients with CAD. Relative risks (RRs) and 95% confidence intervals (95% CI) reflecting the size of these associations are shown; 95% CIs that do not cross 1 denote statistical significance.

**Figure 4.**
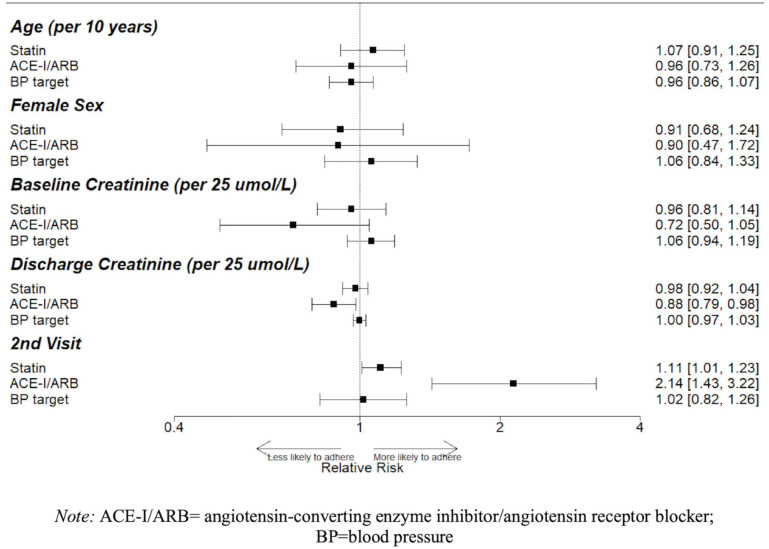
Factors associated with guideline accordance in patients with preexisting CKD. Relative risks (RRs) and 95% confidence intervals (95% CI) reflecting the size of these associations are shown; 95% CIs that do not cross 1 denote statistical significance.

For every 25 μmol/L increase in the discharge serum creatinine, patients with diabetes had a 19% lower relative risk (RR) of being on metformin (95% CI, 7%-29%) and 19% lower RR of being on an ACE-I/ARB (95% CI, 8%-28%), while patients with preexisting CKD had a 12% lower RR of being on an ACE-I/ARB (95% CI, 2%-21%). The second eligible visit was associated with an increased likelihood of being on an ACE-I/ARB across all disease groups, and an increased likelihood of statin therapy for patients with diabetes (RR, 9%; 95% CI, 0%-20%) and preexisting CKD (RR, 11%, 95% CI, 1%-23%). There was no significant association between baseline serum creatinine and use of guideline-based therapy (Figures 2-4).

For the composite score (Table 3), the proportion of eligible recommendations completed decreased by 2% (95% CI, 1%-4%) for every 25 μmol/L increase in the discharge serum creatinine and increased by 18% (95% CI, 11%-26%) for patients with CAD. The second eligible visit was associated with a 3% increase in the composite score but did not reach statistical significance (95% CI, 0%-7%).

**Table 3.** Factors Associated With Guideline-Based Therapy, as Measured by the Composite Score.

Variable	Change in the composite score, % (95% CI)
Age (per 10 years)	−0.3 (−3.8, 3.1)
Female sex	−2.2 (−9.3, 4.9)
Diabetes	−0.4 (−7.6, 6.7)
Coronary artery disease	18.2 (10.6, 25.8)
Baseline serum creatinine (per 25 μmol/L)	−1.0 (−4.5, 2.5)
KDIGO AKI Stage 3 (vs stages 1-2)	2.2 (−4.9, 9.3)
Discharge serum creatinine (per 25 μmol/L)	−2.3 (−3.6, −1.0)
Second eligible visit	3.3 (−0.4, 7.1)

*Note.* AKI= acute kidney injury; CI = confidence interval; KDIGO = Kidney Disease Improving Global Outcomes.

## Discussion

In 213 survivors of AKI followed up in a nephrologist-led post-AKI clinic, we found low utilization of guideline-based therapy in patients with diabetes, CAD, and/or preexisting CKD at 12 to 24 months after an AKI episode. We observed improvements in the usage of ACE-I/ARB in all disease groups at the second eligible post-AKI clinic visit, as well as more statin use in patients with preexisting CKD. Having a second eligible clinic visit was associated with greater use of ACE-I/ARB across all groups. A higher discharge serum creatinine was associated with less use of ACE-I/ARB in patients with diabetes and preexisting CKD, posing the greatest barrier to guideline accordance in our participants. These results suggest that there are key opportunities to improve cardiovascular risk factor management in survivors of AKI, especially for patients with an elevated discharge serum creatinine.

Other studies have also demonstrated low cardiovascular drug usage after an AKI episode. For example, Leung et al^12^ showed in almost 6000 patients who suffered from contrast-associated nephropathy after an acute coronary syndrome that 77% of patients with KDIGO AKI stage 1 and 64% with AKI stage 2-3 were on an ACE-I/ARB within 120 days. In the same study, statins were seen in 81% of patients with stage 1 AKI and only 65% with stage 2-3 AKI.^12^ Brar et al^15^ found that in over 46 000 survivors of AKI, only 48% were using an ACE-I/ARB 6 months after discharge. In a different cohort, the same group saw that only 38% of survivors of AKI were on a statin within 2 years after discharge.^16^ Recent work from the Assessment, Serial Evaluation, and Subsequent Sequelae of Acute Kidney Injury (ASSESS-AKI) prospective cohort study found that the usage of ACE-I/ARB, beta-blockers, and statins 3 months after an AKI episode was 50%, 63%, and 59%, respectively.^31^ Our study adds to this literature by evaluating drug use after AKI in relation to patient-specific evidence-based recommendations, including BP control. We demonstrated that less than 25% of patients followed in a post-AKI clinic met all guideline-based recommendations even 12 to 24 months after AKI, and identified those most susceptible to poorer guideline accordance.

Increase in the use of guideline-based therapy after AKI occurred for ACE-I/ARB therapy between the first and second eligible clinic visits. We saw these improvements even in patients with preexisting CKD. Most patients were not on ACE-I/ARB therapy at their first post-AKI clinic visit, despite this visit occurring approximately 40 days after hospital discharge when between 80% and 90% of patients would have already visited their primary care provider (PCP).^32^ These findings may reflect a number of possible causes. The low use of ACE-I/ARB therapy may be explained by nephrologists having more comfort compared with PCPs in prescribing an ACE-I/ARB after AKI, as there is evidence that PCPs find AKI a complex condition to manage.^33^ Alternatively, nephrology follow-up may have led to better communication between the PCP and the rest of the medical team, who were then prompted to start relevant medications and felt more comfortable to do so in a shared-care approach. It is also possible that these patients had PCP or alternate specialist follow-up between the post-AKI clinic visits, and the ACE-I/ARB therapy was started/restarted there. Statin usage in patients with preexisting CKD also improved. Along with the reasons mentioned above, this may be due to more PCP comfort given their limited side effect profile or nephrologists having a better awareness of CKD being a risk factor for CVD. As there is no comparison group in our study, our results are meant to be hypothesis generating and inform future research and quality improvement initiatives in this area.

CAD was the strongest predictor of an improvement in the composite score. Although we did not collect data on cardiology follow-up, prior work suggests 70% of patients who survive a myocardial infarction (MI) see a cardiologist within 3 months of discharge.^34,35^ This co-management may have contributed to better guideline accordance in this group. Patients and physicians may also have a better awareness of the symptoms of CAD and the need for risk factor modification, as chest pain is a common presenting complaint in emergency departments and PCP offices.^36^

One area where we did not find an improvement between clinic visits was BP control. There is little data to guide short-term BP targets in survivors of AKI. Hypotension is a known risk factor for AKI, and intensive systolic BP lowering is associated with more AKI episodes and incident CKD.^37-39^ For these reasons, there may have been concerns about lowering the BP soon after a hospitalization with AKI, especially when evidence showing improved cardiovascular outcomes with a systolic BP of less than 120 mm Hg had yet to be published.^40^ Given that an AKI episode has been found to be an independent risk factor for the development of hypertension and cardiovascular disease,^41^ more research is needed to determine both BP targets and how aggressively it should be lowered after an episode of AKI.

We did not evaluate the usage of sodium-glucose cotransporter 2 inhibitors or nonsteroidal mineralocorticoid receptor antagonists, which emerged as beneficial medications in trials that were conducted during and after our study.^42-46^ The challenges with using these medications may be similar to those for ACE-I/ARBs, as these medications also contribute to a temporary rise in the serum creatinine and/or hyperkalemia. Consequently, an elevated discharge serum creatinine may also be a barrier to prescribing these medications in survivors of AKI similar to what we observed with ACE-I/ARB therapy. Going forward, these drugs and their evidence-based indications create another opportunity for post-AKI clinics to support guideline accordance for cardiovascular risk reduction.

Strengths of this study include its multicentre design at 2 hospitals with expertise in post-AKI care and the presence of an active intervention targeting survivors of AKI. Since these clinics focused specifically on post-AKI care, the cohort was well characterized in terms of preexisting comorbidities, baseline kidney function, and use of guideline-based therapy over time.

Our study also has limitations. First, the relatively small sample size reduced the number of covariates for which we could adjust, though we included most key factors associated with adverse events after AKI identified by others.^47^ Second, we did not include a comparator group that received usual care, so any benefits cannot necessarily be attributed to the post-AKI clinics. Third, due to the number of comparisons, false positives are possible. However, this work is meant to be hypothesis generating and highlight how nephrologists may be able to support the use of guideline-based therapy after AKI. Fourth, we limited the analysis to patients who had at least 2 clinic visits. While this strengthened our ability to ascertain the use of guideline-based therapy at 12 and 24 months post-AKI, it may have selected for healthier patients more willing to participate in their care. Thus, we may have overestimated the use of guideline-based therapy in our cohort. Fifth, we did not routinely collect data on the reasons why guideline-based therapy was not prescribed. Some of the potential reasons could be related to patient age and frailty, unclear scope of benefit due to prognosis, risk of adverse effects, or lack of knowledge around their benefit. Last, the selected clinical practice guidelines for ACE-I/ARB therapy in patients with CAD and preexisting CKD required some modifications due to incomplete data on baseline left ventricular ejection fraction and proteinuria (data on baseline ejection fraction and proteinuria were missing for 12% and 66% of the cohort, respectively). As a result, we may have overestimated the number of patients eligible for ACE-I/ARB therapy based on clinical practice guidelines. Nonetheless, these patients might still benefit from ACE-I/ARB treatment after AKI given that multiple recent studies have shown an association between ACE-I/ARB usage after AKI and decreased mortality.^15,31,48^

Our study showed that there is low accordance with cardiovascular risk reducing guidelines in AKI survivors with diabetes, CAD, and preexisting CKD. We found that patients with 2 visits in the post-AKI clinic had the greatest use of ACE-I/ARB in all disease groups. We also showed that a higher discharge serum creatinine was associated with lower use of ACE-I/ARB in patients with diabetes and preexisting CKD. One of the possible reasons may be improved comfort amongst physicians in a shared-care model with a nephrologist. This may be particularly relevant in patients with an elevated serum creatinine at hospital discharge who have evidence-based indications for ACE-I/ARB therapy. However, this population faces several challenges to follow-up care after AKI, including low awareness of AKI and its consequences, and competing health demands.^49^ Future work should focus on investigating how much a dedicated post-AKI clinic contributes to guideline accordance relative to primary care and other specialists, as well as on quality improvement interventions that enable high-risk patients and their PCPs to more easily access nephrologists for advice and support on risk factor management.

## Supplemental Material

sj-pdf-1-cjk-10.1177_20543581221103682 – Supplemental material for Use of Guideline-Based Therapy for Diabetes, Coronary Artery Disease, and Chronic Kidney Disease After Acute Kidney Injury: A Retrospective Observational Study Click here for additional data file. Supplemental material, sj-pdf-1-cjk-10.1177_20543581221103682 for Use of Guideline-Based Therapy for Diabetes, Coronary Artery Disease, and Chronic Kidney Disease After Acute Kidney Injury: A Retrospective Observational Study by Sunchit Madan, Patrick A. Norman, Ron Wald, Javier A. Neyra, Alejandro Meraz-Muñoz, Ziv Harel and Samuel A. Silver in Canadian Journal of Kidney Health and Disease
