# Supplementary material for: Use of Guideline-Based Therapy for Diabetes, Coronary Artery Disease, and Chronic Kidney Disease After Acute Kidney Injury: A Retrospective Observational Study
Source: Can J Kidney Health Dis. 2022 Jun 14;9:20543581221103682. doi: 10.1177/20543581221103682 (PMC9201307; doi:10.1177/20543581221103682)
Supplement: sj-pdf-1-cjk-10.1177_20543581221103682 – Supplemental material for Use of Guideline-Based Therapy for Diabetes, Coronary Artery Disease, and Chronic Kidney Disease After Acute Kidney Injury: A Retrospective Observational Study [file sj-pdf-1-cjk-10.1177_20543581221103682.pdf]

**Supplementary Document 1: Acute Kidney Injury Clinic Template - New Patient**

Assessment date: ___________________ Age: _______ Sex: [M] [F]

Ethnicity: ☐ Caucasian ☐ Black African ☐ South Asian ☐ Chinese ☐ Indigenous ☐ Other

**Past Medical History:**

**AKI Cause & Severity:**

Cause of AKI (select all applicable):

☐ ATN ☐ Cardiac Surgery ☐ Cardiorenal Syndrome ☐ Contrast Induced ☐ GI Bleed ☐ Hemolysis
☐ Hepatorenal Syndrome ☐ Hypotension ☐ Medication (specify):_______________________________
☐ Non-Cardiac Surgery ☐ Obstruction ☐ Poisoning ☐ Pre-Renal Azotemia
☐ Rhabdomyolysis ☐ Sepsis ☐ Tubulointerstitial Nephritis ☐ Other:___________________

Baseline Cr: _____________ Date:_________________________________

Peak Cr:________________ Cr at discharge: ______________________ Dialysis: [Yes] [No]

Baseline proteinuria: Date________________________________________

Urine ACR:______________ **OR** Urinalysis: Normal 1+/trace 2+/higher **OR** N/A

**Current Medications (number):**_____________

**Pre-Admission Medications On-Hold:**

**Active Chemotherapy:** [Yes] [No] **NSAIDs:** [Yes] [No] **Allergies:** ________________

| **Family History:** | **Social History:** |
| --- | --- |
| | |

**Symptoms:**

**Physical Exam:**

| | | |
|---|---|---|
| [BP] | [HR] | [Weight] |
| [Volume Status] | | [Other] |

**Labs:** Date_______________

Your patient recently had a hospitalization with acute kidney injury (AKI). Almost 1 in 3 AKI survivors will die within one year of hospital discharge, and AKI is associated with the development of accelerated chronic kidney and cardiovascular disease. Cancer is also a major comorbidity in this patient population. We will follow your patient's kidney function 2-3 times over the next year, and we are happy to assist with management decisions for this high-risk patient population.

**Clinic Impression and Recommendations:**

**On Dialysis:** Yes No If No → **Most recent creatinine**:_________ eGFR:___________

**Most recent urine ACR:**_______________________

**Secondary prevention meds:** [ASA] [Statins] [BB] [ACEi/ARB]

Statins are recommended for most patients with eGFR<60mL/min/1.73m$^2$

Consider stopping all ACE inhibitors or ARBs for 3 months in patients with preserved LVEF

**Drugs stopped:**____________________________ **Drugs started:**______________________________

**Drugs decreased:**___________________________ **Drugs increased**:____________________________

**Procedures ordered:** [Ultrasound] [Echo] [Other]________________________

**Referrals:** [Urology] [Cardio] [Other]________________________

**If Cancer, communicate with oncologist:** a) If ok to use IV contrast b) If chemo needs to be adjusted

**Sick day counselling:** SADMAN → Sulfonylurea, ACE-I, Diuretic, Metformin, ARB, NSAID

**Other:** **Return to Clinic:**
